# *Brucella* infection of the thoracic vertebral arch presenting with an epidural abscess: a case report

**DOI:** 10.1186/s13256-015-0713-6

**Published:** 2015-10-23

**Authors:** ZhiXun Yin, ErXing He, HongMei Ding, JingChen Chen

**Affiliations:** 1 Department of Orthopaedic Surgery, the First Affiliated Hospital of Guangzhou Medical University, No. 151 Yanjiang Road, Guangzhou, Guangdong 510120 China; 2 Guangzhou Medical University, No, 195 Dongfeng Xi Road, Guangzhou, Guangdong 510182 China

**Keywords:** Brucellosis, Epidural abscess, Neural arch, Specific infection

## Abstract

**Introduction:**

Although *Brucella* spondylitis and *Brucella* discitis have been frequently reported, *Brucella* infection of the vertebral arch is rare and has not been previously described. We present the first case of *Brucella* infection of the thoracic vertebral arch with epidural abscess formation and discuss the clinical key points.

**Case presentation:**

A 57-year-old man of Han nationality with a history of contact with an isolated sheep stomach 2 months previously was admitted with an undulant fever, night sweats, back pain, and weakness. Thoracic magnetic resonance imaging showed laminar destruction of T9 and an epidural abscess at the T9 to 10 level with significant cord compression. Diagnosis of *Brucella* infection of his vertebral arch was confirmed by a positive blood culture with growth of *Brucella melitensis*. Total laminectomy, abscess cleansing, and percutaneous pedicular screw fixation was performed initially, followed by antibiotic treatment with a combination of doxycycline and rifampin for 4 months. Recovery was confirmed by clinical, magnetic resonance imaging, and blood culture findings.

**Conclusions:**

This is an unusual case of *Brucella* infection of the vertebral arch with epidural abscess formation. Effective antibiotic therapy of a sufficient duration and timely performance of surgical treatment are the key points in management of such cases.

## Introduction

Brucellosis is an endemic and systemic disease with characteristic symptoms of undulant fever, night sweats, and weakness [1]. It can involve any organ or tissue, including the eyes, liver, lungs, nervous system, cardiovascular system, bone, and joints [2]. Localized brucellosis most commonly involves the bones and joints (10 to 80 % of cases) [1, 3], especially the axial skeleton, and results in *Brucella* spondylitis, sacroiliitis, and peripheral arthritis. In the spine, the vertebral bodies and intervertebral disk areas are the most frequently affected sites [4], and *Brucella* spondylitis or discitis with epidural abscess formation has been frequently reported [4, 5]. However, the posterior arch of the vertebra is rarely involved, and no such cases have been reported.

We present an unusual case of brucellosis with neural arch infection and epidural abscess formation at the thoracic level. The case is special not only because brucellosis involving the posterior arch of the vertebra is rare, but also for the different pathological changes and operative methods.

## Case presentation

**Fig. 1:**
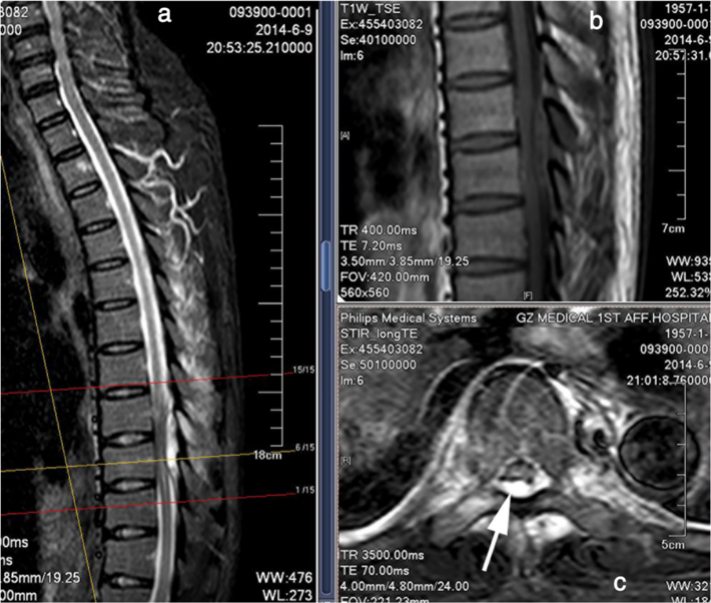
Magnetic resonance imaging of the thoracic vertebra. **a** Contrast-enhanced T2-weighted sagittal scan demonstrates a high-signal lesion behind the dural sac at the T8–9 level. **b** T1-weighted sagittal scan shows that the lesion has an area of moderate signal intensity and low-signal spots. **c** T2-weighted axial scan shows a high-signal area in both the vertebral canal and paravertebral muscle. The epidural abscess (single-headed arrow) is behand the spinal cord and dural sac

**Fig. 2:**
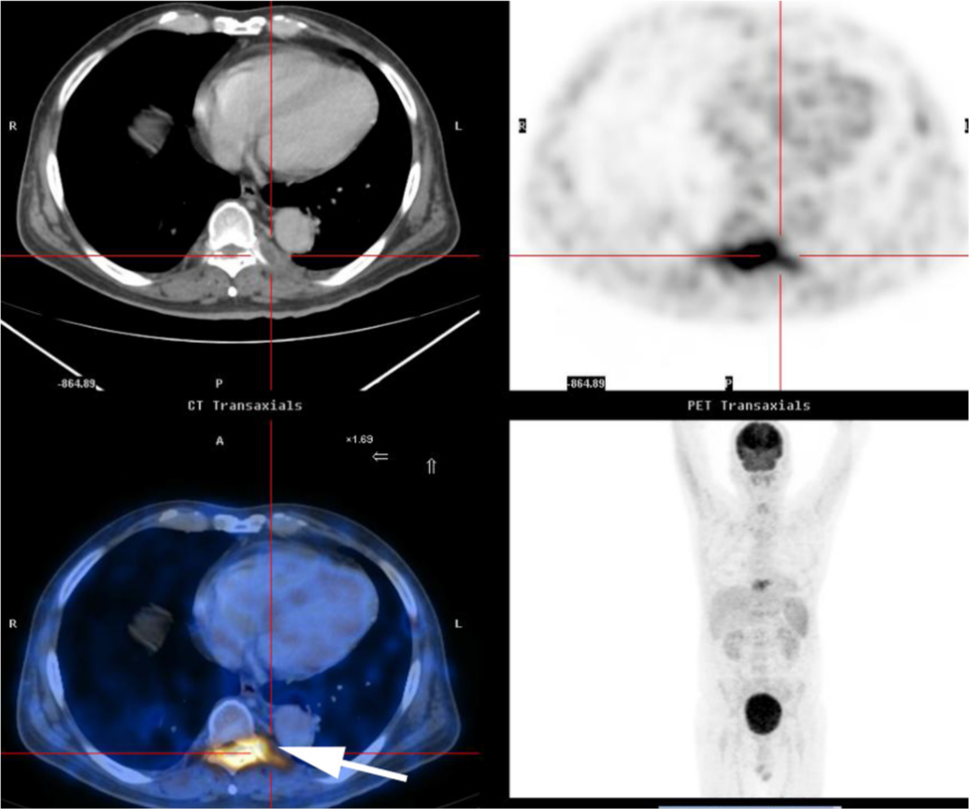
Positron emission tomography-computed tomography scan of the total body. Radionuclide concentration (single-headed arrow) was detected at the vertebral arch of T9

A 57-year-old man of Han nationality with a 1-month history of high fever, night sweats, and back pain was admitted to the Respiratory Department of the First Affiliated Hospital of Guangzhou Medical University in June 2014. Before admission he had consulted with the Chinese Medical Hospital of Guangdong Province where he was treated with cefprozil tablets for a fever. He had touched an unpasteurized isolated stomach of a sheep with his injured hand about 1 month prior to admission to our hospital. The fever was initially presumed to be secondary to tuberculosis or a metastatic tumor. Routine blood testing revealed a white blood cell count of 7170/mm^3^ (normal, 4000 to 10000/mm^3^) with 80 % neutrophils (normal, 40 to 70 %), erythrocyte sedimentation rate of 35mm/hour (normal, 0 to 20mm/hour), C-reactive protein level of 3.19mg/dL (normal, 0to 0.6mg/dL), and procalcitonin level of 0.21ng/L (normal, 0 to 0.05ng/mL). A serological test for tuberculosis (Hexagon TB; HUMAN Diagnostics, Wiesbaden, Germany) was negative. The positive blood test indicated he had an infection. On the sixth day after admission, a Gram-negative bacillus was detected in his blood culture, and Sulperazone (sulbactam and cefoperazone) had already been administered intravenously. On the same day, he experienced weakness and numbness in both legs and difficulty urinating, but his temperature decreased to normal. A physical examination performed by an orthopedist revealed grade 3/5 paraparesis in his right lower limb, grade 4/5 paraparesis in his left lower limb, and hypermyotonia in both lower limbs, and positive percussion and palpation at the spinous processes of T9 and 10. No other pathological findings were detected. Thoracic magnetic resonance imaging (MRI) was immediately conducted, and a laminar inflammatory reaction of T9 and rear epidural abscess between T9 and 10 were found; significant cord compression was observed at these levels (Fig. 1). Positron emission tomography-computed tomography (CT) revealed osteolytic destruction with radionuclide steps at the arch of T9 (Fig. 2). We presumed that he had a specific infection in the neural arch of his thoracic vertebra and he was transferred to the Department of Orthopedics for an operation the next day.

After 1 day of preoperative preparation, he underwent total laminectomy at T8 to 10 with percutaneous pedicle screw fixation at T7 to 8 and T11 to 12. The abscess and lesion (composed of sequestrum fragments, inflammatory granulation tissue, and smaller separate abscesses) were removed after collecting samples for pathology and culture. Chronic nonspecific inflammation was detected in the biopsy sample, and no bacteria grew in the culture. One day after the operation, the previously cultured Gram-negative bacillus from his blood was identified as *Brucella melitensis*, and a with minimal inhibitory concentration (MIC) of 0.094ug/ml, and rifampin was also sensitive with 26 in Kirby–Bauer (KB) method. Thus, he was finally diagnosed with *Brucella* infection of the thoracic vertebral arch with epidural abscess formation. He received antibiotic therapy with cefoperazone sodium and sulbactam sodium (1.5g twice daily, since admission) administered intravenously for 4 weeks and antibiotic therapy with doxycycline (100mg twice daily) and rifampin (450mg once daily) administered orally for 4 months according to the drug sensitivity test. After this treatment, his recovery was confirmed by clinical findings (disappearance of night sweats, weakness, and fever), laboratory test results (three negative blood cultures), and MRI findings (adequate decompression, no abscesses, and no inflammatory reaction in his spine). He was discharged 4 weeks after the operation and followed up in our out-patient clinic for 10 months with no evidence of disease recurrence. He has also been followed up in the Epidemic Prevention Station of Guangzhou city; the Rose Bengal test was negative in his serum three times together with a tube agglutination titer of 1/24.

## Discussion

Brucellosis is an endemic and zoonotic disease caused by Gram-negative bacteria of the genus *Brucella* [1]. *Brucella* species are transmitted directly or indirectly from infected animals to humans. The genus *Brucella* was first discovered by David Bruce in 1887 [6]. Since the report of human brucellar spondylitis by Kulowski and Vinke in 1932 [7], there have been several case reports about spinal brucellar infection. In 1987, Goodhart *et al*. [8] reported the case of a patient with spinal brucellosis with bilateral paraspinal abscesses. Subsequently in 1999, Pina *et al*. [9] presented a case of *Brucella* spondylodiscitis and an epidural abscess in the cervical spine. That same year, Bingol *et al*. [10] presented a case of a thoracic intramedullary *Brucella* granuloma that was resolved by medication. In 2000, Zormpala *et al*. [11] reported a case of *Brucella* spondylitis involving both the cervical and lumbar spine. In 2007, Cobbaert *et al*. [12] encountered an uncommon case of *Brucella* spondylodiscitis in the lumbar spine in a patient with abdominal pain. Also in 2007, Nas *et al*. [13] were the first to report a case of an intramedullary *Brucella* granuloma in the cervical spine. Finally, in 2010, Yilmaz *et al*. [14] described a patient with a lumbar disc herniation caused by *Brucella* discitis. Approximately 500,000 cases of brucellosis are reported annually worldwide, most of which occur in developing countries [1, 2]. Although there were lots of reports about brucellar infection involving the vertebral body or intervertebral disc, there was no previous report about brucellar infection involving the vertebral arch.

**Fig. 3:**
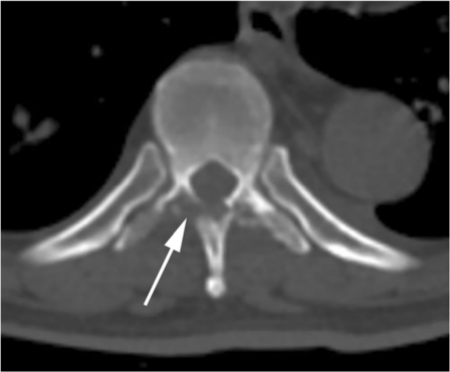
Computed tomography scan of the thoracic vertebra. A destructive focus (single-headed arrow) in the bilateral vertebral plates and transverse processes was detected

The clinical features of spinal brucellosis depend on various factors including the size of the infected area, route of infection, age of patient, duration of the disease, and *Brucella* species [15]. This case had a similar presentation to previously published cases of spinal brucellosis (for example, spondylitis and spondylodiscitis) [7, 9, 12]. These clinical features are often manifestations of chronic infection, such as undulant fever, night sweats, malaise, and anorexia; a physical examination often reveals hepatomegaly and splenomegaly. Localized symptoms (for example, back pain, intercostal pain), localized signs (for example, tenderness on palpation), and sometimes neurological deficits (for example, sensory, motor, and reflex changes secondary to nerve root or spinal cord compression) can also be detected [4]. Fortunately, the radiological signs of neural arch brucellosis are very different from those of *Brucella* spondylitis or spondylodiscitis, helping doctors to establish differential diagnoses among them. CT and MRI allow for prompt detection of the position of the bony destructive focus, the extent of the inflammatory process, and the location of the epidural abscess (Figs. 1 and 3). In patients with neural arch brucellosis, the bony destructive focus is located on the neural arch (for example, vertebral plate, spinous process, or transverse process), the inflammatory region mainly involves the local paravertebral muscles, and the epidural abscess is positioned behind the dural sac. In patients with *Brucella* spondylitis, the bony destructive focus and inflammatory area are located on the vertebrae, while in patients with *Brucella* spondylodiscitis they are located on the vertebral endplate and intervertebral disc. The epidural abscess is positioned before the dural sac in both *Brucella* spondylitis and spondylodiscitis.

The diagnosis of spinal brucellosis is mainly confirmed by clinical features, imaging examination of the spine, and laboratory findings of brucellosis (positive serum agglutination and/or blood culture) [4]. In the present case, the clinical features were obvious with undulant fever, night sweating, back pain, progressive weakness, and hypermyotonia in both legs. CT and MRI revealed a laminar osteolytic focus and inflammatory reaction in T9 and an epidural abscess with significant cord compression between T9 and 10. A blood culture was positive for *B. melitensis*.

This specific case of neural arch brucellosis was treated similar to other types of spinal brucellosis with a good outcome. Antibiotic treatment is one of the keypoints in dealling with brucellosis; the most common antibiotic treatment is a combination of doxycycline (100mg every 12 hours orally) and rifampicin (600 to 900mg/day orally) for 8 weeks or more [4]. Many researchers have emphasized that the duration of antibiotic therapy should be based on the patient’s individual condition and the extent of the lesions [4, 5, 15]. Postoperatively, our patient was cured by a combination of doxycycline (100mg every 12 hours orally) and rifampicin (600mg/day orally) for 8 weeks, and no relapse was detected in follow-ups over 1 year. In this case, Sulperazone (sulbactam and cefoperazone) was used for 4 weeks after admission, at the sixth day of using Sulperazone (sulbactam and cefoperazone) the fever was controlled so we believed that Sulperazone (sulbactam and cefoperazone) is sensitive in brucellar infection. Unfortunately, Sulperazone (sulbactam and cefoperazone) was not included in the drug sensitivity test.

The need for a surgical operation in patients with spinal brucellosis [4] is considered in cases of spinal instability, cord compression, radiculopathy, cauda equina syndrome, and epidural abscess formation. Surgical intervention was performed in this patient to treat cord compression caused by the epidural abscess. On intraoperative examination we found many small abscesses separated by fibrous granulation tissue; however, no dissociative sequestra were found in either the abscess or fibrous granulation tissue, differentiating it from spinal tuberculosis.

This study has two notable limitations. The most significant is the lack of serous agglutination in the first 3 months of the patient’s clinical course of illness. In addition, the follow-up period was short; longer observation periods are needed to definitively exclude the presence of recurrence.

## Conclusions

This case of *Brucella* infection of the vertebral arch with epidural abscess formation is unusual. Effective antibiotic therapy for a sufficient duration and timely performance of surgery are the key points to consider in managing such cases. We believe that Sulperazone (sulbactam and cefoperazone) is sensitive in brucellar infection.

### Patient’s perspective

I have no medical knowledge about my case and only write from my own perspective and experience to provide assistance to the case report.

When I was 57 years of age, I had a full-time job at a private enterprise. I believed that I was in very good health; I had never been admitted to hospital for any problems. However, on one afternoon last summer I became very exhausted and hot. I thought I must have had influenza, so I drank more water than usual, rested in bed, and took Bufferin (aspirin) twice a day. When I awoke the next morning I no longer felt hot, but instead experienced cool sweats. That afternoon I felt worse and went to see a doctor in a traditional Chinese medicine hospital in Guangzhou. A nurse took my temperature, which was 38.7 °C, and introduced me to the doctor. The doctor asked me if I had a headache or backache. I said yes and felt very tired. After a routine blood test I was determined to have a fever of unknown origin and was given cefprozil tablets and treatment with traditional Chinese medicine. A nurse gave an injection in my arm, but I don’t know what the injection was. Whatever it was, it relieved the fever instantly. I followed the prescribed therapy at home for nearly 1 month, but still ran a temperature, although it was below 38 °C most afternoons; it was relieved at night. The therapy did not seem to be effective; my backache worsened, I developed progressive weakness, and I had a slight cough. Therefore, I visited a professor in the Department of Respiration of the First Affiliated Hospital of Guangzhou Medical University. He presumed that I may have tuberculosis and advised me to undergo further testing and treatment in his hospital.

I was admitted to the hospital in June 2014. They asked me if I had been in contact with any people with tuberculosis; I said no, there was little chance of this. They then asked me if I had been in contact with any animals. I remembered that I had touched the isolated stomach of a sheep with my injured hand about 1 month previously. Many blood tests and some X-rays, CT, and MRI were performed, and intravenous infusions and antibiotics were administered. I didn’t run a fever for 5 days after admission, but I felt weak and numb in both legs, and it was difficult to walk and urinate. On the sixth day after admission, I was informed that MRI showed evidence of a specific infection in the thoracic spine, and I was transferred to the Department of Orthopaedic Surgery the next day. An operation was performed 8 days after admission. The next day I was diagnosed with brucellosis based on a positive blood culture and treated with doxycycline and rifampin for at least 4 months.

After nearly a month of hospitalization, I spent approximately 4 months taking oral antibiotics at home with doxycycline and rifampin. Three months after leaving the hospital, I had a good appetite for strongly flavored food, no longer felt tired, and experienced no further night sweats. During this period, I underwent blood cultures and agglutination tests three times, and all results were negative. I underwent another MRI examination in September, and no compression or abscesses were detected in my spine. My doctor informed me that I had completely recovered. I felt fully back to health and had returned to work by the end of October. After a further 8 months of follow-up, I had no evidence of recurrence.

## Consent

Written informed consent was obtained from our patient for the publication of this case report and any accompanying images. A copy of the written consent is available for review by the Editor-in-Chief of this journal.
